# Vascular endothelial growth factor suppresses TNFSF15 production in endothelial cells by stimulating miR‐31 and miR‐20a expression via activation of Akt and Erk signals

**DOI:** 10.1002/2211-5463.12171

**Published:** 2016-12-28

**Authors:** Hui‐Ting Deng, Hai‐Lin Liu, Bei‐Bei Zhai, Kun Zhang, Guo‐Ce Xu, Xue‐Mei Peng, Qiang‐Zhe Zhang, Lu‐Yuan Li

**Affiliations:** ^1^ State Key Laboratory of Medicinal Chemical Biology and College of Pharmacy, and Tianjin Key Laboratory of Molecular Drug ResearchNankai UniversityChina; ^2^ Collaborative Innovation Center for BiotherapyNankai UniversityWest China HospitalSichuan UniversityChengduChina

**Keywords:** Akt, Erk, miRNA, TNFSF15, VEGF

## Abstract

Tumor necrosis factor superfamily‐15 (TNFSF15; VEGI; TL1A) is a negative modulator of angiogenesis for blood vessel homeostasis and is produced by endothelial cells in a mature vasculature. It is known to be downregulated by vascular endothelial growth factor (VEGF), a major regulator of neovascularization but the mechanism of this interaction is unclear. Here we report that VEGF is able to stimulate the production of two microRNAs, miR‐20a and miR‐31, which directly target the 3′‐UTR of TNFSF15. Additionally, we show that two VEGF‐stimulated cell growth signals, Erk and Akt, are responsible for promoting the expression of miR‐20a and miR‐31. Treatment of human umbilical vein endothelial cells (HUVECs) with Akt inhibitor LY294002 results in diminished miR‐20a and miR‐31 production, while Erk inhibitor U0126 prevented VEGF‐stimulated expression of miR‐20a but not that of miR‐31. Furthermore, inactivation of either Erk or Akt signals restores TNFSF15 gene expression. In an angiogenesis assay, elevated miR‐20a or miR‐31 levels in HUVECs leads to enhancement of capillary‐like tubule formation *in vitro*, whereas lowered miR‐20a and miR‐31 levels results in an inhibition. These findings are consistent with the view that miR‐20a and miR‐31 mediate VEGF‐induced downregulation of TNFSF15. Targeting these microRNA molecules may therefore provide an effective approach to inhibit angiogenesis.

**Abbreviations**
EC: endothelial cell
HUVECs: human umbilical vein endothelial cells
miRNA or miR: microRNA
TNFSF15: tumor necrosis factor superfamily‐15
VEGF: vascular endothelial growth factor

Tumor necrosis factor superfamily‐15 (TNFSF15, also known as VEGI or TL1A) is a cytokine produced predominantly by endothelial cell (EC) in established blood vessels, and is a specific inhibitor of EC proliferation 1. TNFSF15 appears to function in the maintenance of vascular homeostasis, being able to enforce growth arrest on quiescent EC but induce apoptosis in proliferating EC 2. Systemic administration of recombinant TNFSF15 results in an inhibition of tumor angiogenesis and tumor growth in animal models 3. It also inhibits Lin^−^‐Sca‐1^+^ endothelial progenitor cell (EPC) differentiation into EC 4 and EPC incorporation into tumor vasculature in murine models 5. However, TNFSF15 expression is absent or marginal in tumor vasculatures in various cancers 6, 7, 8.

Chronic inflammation such as that observed in cancers, fueled by periodic hypoxic conditions, disrupted vascular integrity, lymphocyte infiltration, and continual generation of dysfunctional blood vessels, is a recognized risk factor for malignancies 9, 10, 11, 12, 13, 14. Cancer cells in hypoxia produce a number of protein factors to promote angiogenesis, including vascular endothelial cell growth factor (VEGF) 15, 16. We reported previously that TNFSF15 downregulation in ovarian cancer is facilitated by VEGF secreted by cancer cells 8. However, the mechanism between VEGF and TNFSF15 remains unclear.

MicroRNA molecules are a class of small, noncoding RNA containing 18–22 nucleotides capable of binding to the 3′‐untranslated region (3′UTR) of mRNA molecules. The interactions result in degradation and transcriptional suppression of the target mRNA 17, 18. Increasing evidences show that miRNAs play a key role in many biological processes, such as cell proliferation, survival, angiogenesis, and cancer cell invasion and metastasis 19, 20.

In this study, we show that two miRNA molecules, miR‐20a and miR‐31, are involved in the downregulation of TNFSF15 gene expression by VEGF. In addition, we demonstrate that VEGF stimulates the production of miR‐20a and miR‐31 by activating the Akt and Erk signaling pathways. Our findings bring forward insights into the balancing mechanisms in the modulation of vascular homeostasis.

## Materials and methods

### Cell culture

The human umbilical vein endothelial cells (HUVECs) were isolated from umbilical cord vein by collagenase treatment. The freshly obtained cells were centrifuged at 300 ***g***, 10 min and resuspended with EGM2 (LONZA, Allendale, NJ, USA) and then plated in flasks covered with gelatin. The cells were incubated at 37 °C and 5% CO_2_ atmosphere overnight. Replicated cultures were obtained by trypsin and were used at passages <5.

### ELISA

Tumor necrosis factor superfamily‐15 concentration in media was detected by ELISA with ELISA kit (PEPROTECH, Rocky Hill, NJ, USA). The 96‐well plates were coated with antibody according to the manufacturer's protocol. The provided standard was used to construct standard curve. Finally, the OD were detected by microplate reader in 450 nm with an iMark^TM^ Microplate Reader (Bio‐Rad Laboratories, Hercules, CA, USA).

### Luciferase reporter assay

To test the direct binding sequence of miR‐20a and miR‐31, the dual‐luciferase reporter assays were constructed. A sequence containing the presumed miR‐20a‐ and miR‐31‐binding site was designed from the TNFSF15 3′UTR. The sequence was inserted into the pmir‐GLO dual‐luciferase miRNA target expression vector. For the luciferase assay, the 293T cells were cultured and seeded in 24‐well plates. Each well was cotransfected with 0.5 μg plasmid and 50 pmol miRNA mimics or miRNA negative control using RNAiMAX Lipofectamine 2000 (Invitrogen, Carlsbad, CA, USA). After 24 hours, the cells for luciferase assay were analyzed with luciferase assay kit (Promega, Madison, USA) according to the manufacturer's instructions.

### RNA isolation and real‐time PCR

Total RNA and miRNA was extracted using miRNA isolation kit according to the manufacturer's protocol which was purchased from Qiagen (Valencia, CA, USA). The cDNA was obtained using Transcript First‐strand cDNA synthesis super‐Mix kit (Transgene, Beijing, China) following the manufacturer's instructions. The following quantitative real‐time PCR was performed using SYBR green supermix (Transgene). miR‐506‐5p RT‐primer (#ssD1251083353), miR‐506‐5p Forward primer (#ssD1251083354), miR‐124‐3p RT‐primer (#ssD809230054), miR‐124‐3p Forward primer (#ssD809230746), miR‐190b RT‐primer (#ssD809230203), miR‐190b Forward primer (#ssD809230895), miR‐145‐5p RT‐primer (#ssD809230156), miR‐145‐5p Forward primer (#ssD809230848), miR‐150‐5p RT‐primer (#ssD809230169), miR‐150‐5p Forward primer (#ssD809230861), miR‐181a‐5p RT‐primer (#ssD809230183), miR‐181a‐5p Forward primer (#ssD809230875), miR‐320a RT‐primer (#ssD809230295), miR‐320a Forward primer (#ssD809230987), miR‐125a‐5p RT‐primer (#ssD809230074), miR‐125a‐5p Forward primer (#ssD809230074), miR‐106a‐5p RT‐primer (#ssD809230015), miR‐106a‐5p Forward primer (#ssD809230707), miR‐24‐3p RT‐primer (#ssD809230262), miR‐24‐3p Forward primer (#ssD809230954), miR‐20a‐5p RT‐primer (#ssD809230237), miR‐20a‐5p Forward primer (#ssD809230929), miR‐31‐5p RT‐primer (#ssD809230293), miR‐31‐5p Forward primer (#ssD1210179103), miR‐Reverse Primer (#ssD089261711), U6‐RT Primer (#ssD0904071008), U6‐Forward Primer (#ssD0904071006), and U6‐Reverse Primer (#ssD0904071007) were purchased from Ribobio (Guangzhou, China). The primers to detected TNFSF15 were 5′‐TTAGA GCAGA CGGAG ATA‐3′ and 5′‐TTGTT GGTAT AGTTC ATTCG‐3′, GAPDH were 5′‐GTCTC CTCTG ACTTC AACAG CG‐3′ and 5′‐ACCAC CCTGT TGCTG TAGCC AA‐3. The miRNA U6 and GAPDH were used for normalization. Real‐time data were analyzed using cycle threshold (delta delta *C*_t_) method.

### *In vitro* angiogenesis assay

Human umbilical vein endothelial cells were plated in 24‐well plates and cultured for 12 h. The cells were transfected with miRNA negative control, miR‐20a mimic, and miR‐31 mimic. Another group was transfected with miRNA inhibitor negative control, anti‐miR‐20a, anti‐miR‐31, and this group was treated with VEGF to improve the expression level of the miRNAs. Four hours later, the HUVECs were digested and plated in 48‐well plates with 50 μL solidified Matrigel and incubated at 37 °C for 9 h. The cells were stained with 3 μm calcein‐AM (Invitrogen) for 30 min at 37 °C and 5% CO_2_. Formation of the capillary‐tubule structures was observed and digitally photographed under an inverted light microscope at 5× magnification (Axiovert 200M; Zeiss, Oberkochen, Germany). Tube lengths and areas were quantified using image‐pro plus 6.0 software (Media Cybernetics, Rockville, MD, USA).

### Western blot analysis

The cells were lysed by RIPA buffer supplemented with protease inhibitor. After cell lysis, the lysates were centrifuged at 13 500 ***g*** for 20 min. The proteins were quantified using BCA (Bicinchoninic Acid), and were run on 12% sodium dodecyl sulfate‐polyacrylamide gel electrophoresis (SDS/PAGE) gel, followed by wet‐transfer process utilizing polyvinylidene fluoride (PVDF) membrane (Roche Molecular Biochemicals, Quebec, Canada). PVDF membrane was then blocked with 5% skim milk powder at room temperature for 1 h. The samples were subject to relevant primary antibodies at 4 °C overnight and then incubated with appropriate HRP‐conjugated secondary antibodies. The films were developed with the ECL System (Millipore, Billerica, MA, USA). TNFSF15 antibody (#sc‐32945, 1 : 1000) was purchased from Santa Cruz Biotechnology (Santa Cruz, CA, USA). Other antibodies for Phospho‐akt (#12178s, 1 : 2000), Akt (#4691s, 1 : 2000), Phospho‐erk (#4370p, 1 : 2000), Erk (#3316s, 1 : 2000) were purchased from Cell Signaling Technology (Danvers, MA, USA).

### Statistical analysis

At least three repeats were carried for each experiment. Data were analyzed with graphpad prism 5.0 (La Jolla, CA, USA) software, following by using the two‐tailed, unpaired Student's *t*‐test or one‐way ANOVA. *P* ≤ 0.05 was considered statistically significant.

## Results

### VEGF downregulates TNFSF15 production in HUVECs

**Figure 1:**
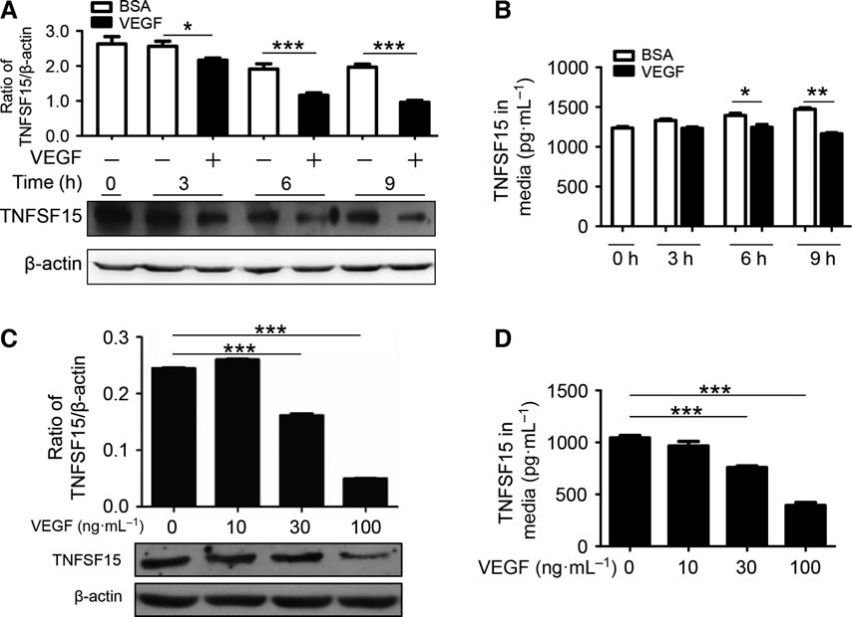
Vascular endothelial growth factor downregulates TNFSF15 in HUVECs. (A) TNFSF15 protein levels in vehicle‐ (white) or VEGF‐treated (black) HUVECs at indicated time intervals. (B) Concentrations of TNFSF15 in culture media were determined by ELISA (*n* = 4) following treatment with BSA (white bar) or VEGF (black bar) at 100 ng·mL^−1^. (C) Changes in TNFSF15 protein levels in HUVECs treated with VEGF at indicated concentrations. (D) TNFSF15 concentrations in culture media were determined by ELISA (*n* = 4) at indicated VEGF concentrations. Data are mean ± SD. *P < 0.05; **P < 0.01; ***P < 0.001; Student's *t*‐test or one‐way ANOVA.

We determined TNFSF15 protein levels in HUVECs in response to VEGF treatment. Using western blot analysis, we found that TNFSF15 protein levels decreased in a time‐dependent manner following addition of VEGF (100 ng·mL^−1^) to the cell cultures (Fig. 1A). ELISA analysis indicated that TNFSF15 concentration in the culture media decreased as well (Fig. 1B). In addition, we found that inhibition of TNFSF15 production, measured either as cell‐associated protein or as a secreted protein in the conditioned media, in HUVECs by VEGF was also dose‐dependent (Fig. 1C,D).

### VEGF upregulates miR‐20a and miR‐31 expression level in HUVECs

**Figure 2:**
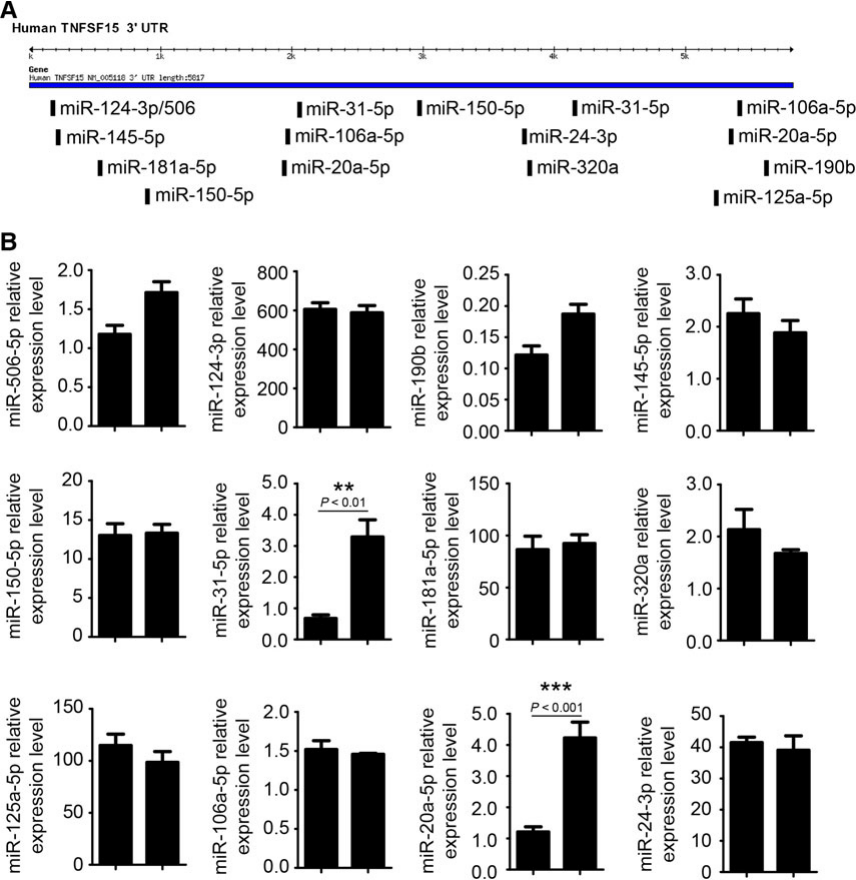
Vascular endothelial growth factor upregulates miR‐20a and miR‐31 expression level in HUVECs. (A) Twelve kinds of high score miRNAs target to the 3′UTR of TNFSF15 by bioinformatics prediction. (B) The changes of 12 kinds of predicted miRNAs using QPCR (*n* = 4). Data are mean ± SD. **P < 0.01; ***P < 0.001; Student's *t*‐test.

We carried out data mining, using Targetsacan Human database (http://www.targetscan.org/) 21, 22, 23, and found 79 miRNAs that may target TNFSF15 transcripts. Additionally, about 200 miRNAs were reported to be expressed in EC 24. An inspection of the 3′‐untranslated region (3′‐UTR) of TNFSF15 gene transcript, we obtain 12 high score miRNA which target to the 3‐UTR of TNFSF15 (Fig. 2A). We therefore determined whether the concentrations of these miRNA molecules in HUVECs would change in response to VEGF treatment of the cells. By using real‐time PCR analysis, we found that two of these miRNA molecules, namely miR‐20a and miR‐31, markedly increased in concentrations by five‐ and threefold, respectively (Fig. 2B). These data demonstrate that VEGF is able to downregulate TNFSF15 production in HUVECs and inhibition of TNFSF15 by VEGF may be achieved by an upregulation of expression levels of miR‐20a and miR‐31.

### TNFSF15 is a direct target of miR‐20a and miR‐31 in HUVECs

**Figure 3:**
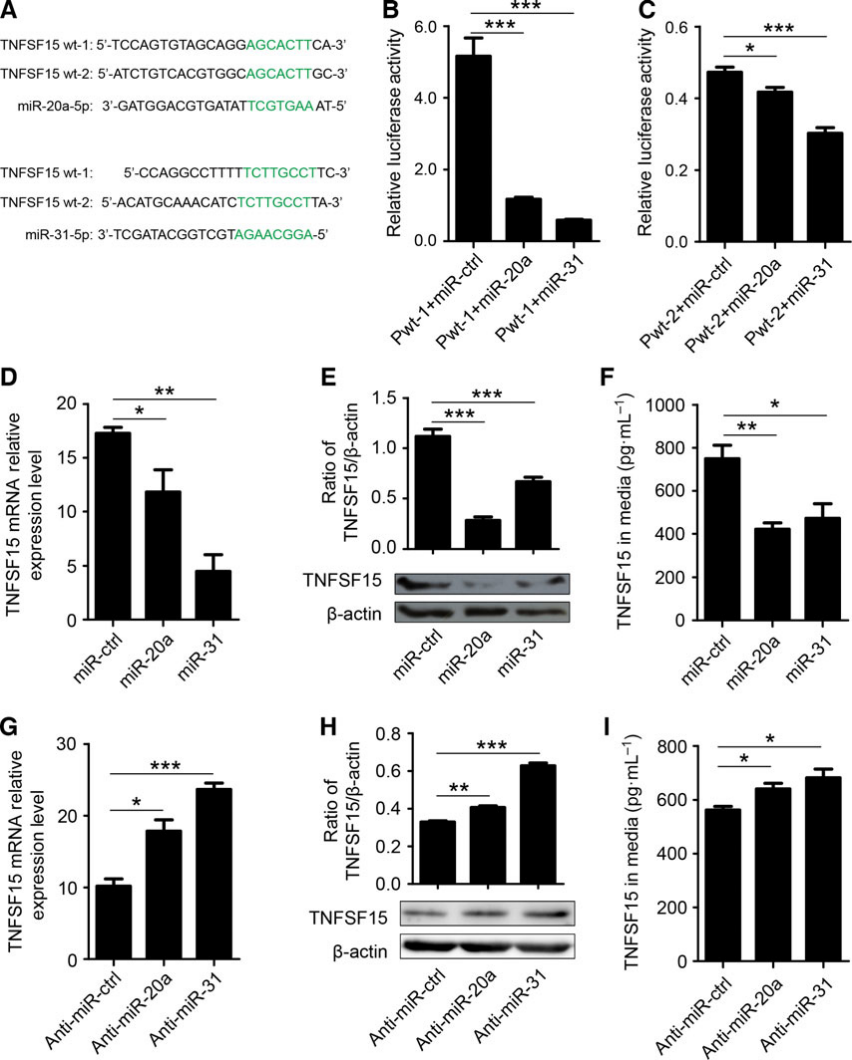
Tumor necrosis factor superfamily‐15 is a direct target of miR‐20a and miR‐31 in HUVECs. (A) Sequences of miR‐20a and miR‐31; Seed sequence and the complementary binding sites are in green. (B, C) Regulation of TNFSF15 by miR‐20a and miR‐31 was confirmed by luciferase reporter; Pwt‐1 and Pwt‐2 indicate two constructed plasmids including proposed binding sites for miR‐20a and miR‐31. (D–F) Changes of TNFSF15 in HUVECs transfected with miR‐20a and miR‐31 mimics; The expression level of TNFSF15 was detected using QPCR (D), western blot (E), and ELISA (F). (G–I) Changes of TNFSF15 in HUVECs transfected with anti‐miR‐20a and anti‐miR‐31; TNFSF15 expression level was detected using QPCR (G), western blot (H), and ELISA (I). Data are mean ± SD.*P < 0.1, **P < 0.01, ***P < 0.001; one‐way ANOVA.

We then determined whether miR‐20a and miR‐31 were able to target the 3′‐UTR of the TNFSF15 transcript. We cloned two fragments of the TNFSF15 3′‐UTR that were complementary to the conserved sequence of miR‐20a and miR‐31 into a dual report plasmid encoding the luciferase report gene (Fig. 3A). We then transiently cotransfected the TNFSF15 3′‐UTR‐luc (pWT1 or pWT2) and the miRNA mimics (miR‐20a‐5p or miR‐31‐5p) in 293T cells, using a scrambled miRNA as a control (miR‐ctrl). We found that both miR‐20a and miR‐31 significantly inhibited the expression of the luciferase reporter compared with the miR‐ctrl (Fig. 3B,C). Additionally, we transfected the miRNA mimics and anti‐miRNA into HUVECs and analyzed the impact on the TNFSF15 expression at both mRNA and protein levels. We found that treatment of the cells with either miR‐20a or miR‐31 resulted in a significant decline of TNFSF15 gene expression, measured as mRNA, cell‐associated protein, or secreted protein in comparison with the results of miR‐ctrl treatment (Fig. 3D–F). Furthermore, we found that transfection of HUVECs with either anti‐miR‐20a or anti‐miR‐31 led to markedly increased expression of the TNFSF15 gene at both mRNA and protein levels compared with that found with the negative control groups (Fig. 3G–I). These findings indicate that the 3′‐UTR of TNFSF15 is a direct target of miR‐20a and miR‐31, and that miR‐20a and miR‐31 is capable of negatively modulating TNFSF15 gene expression.

### miR‐20a and miR‐31 downregulate TNFSF15 expression through activation of the Akt and Erk signaling pathways

**Figure 4:**
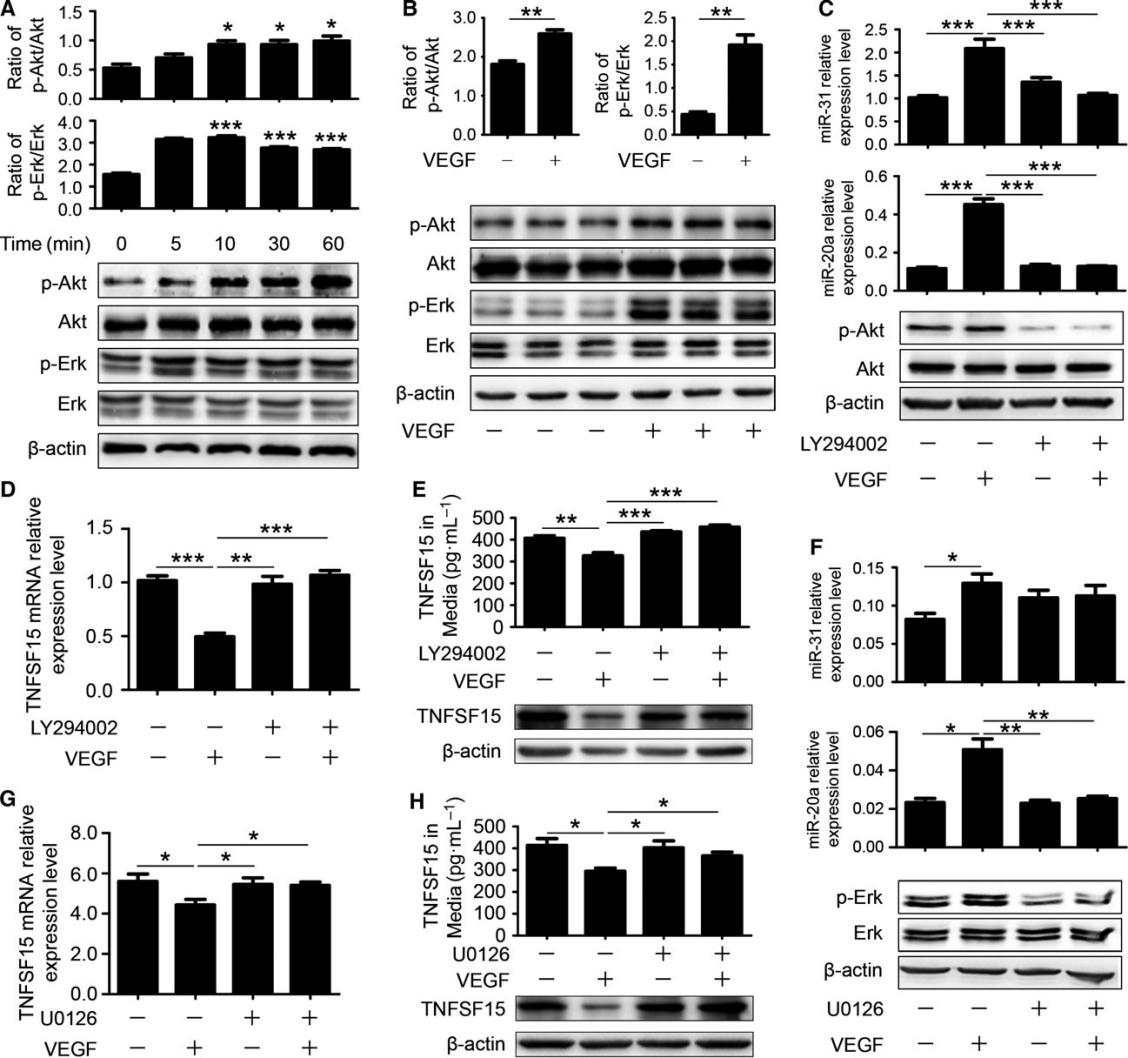
miR‐20a and miR‐31 downregulate TNFSF15 expression through activation of the Akt and Erk signaling pathways. (A) Western blot analysis of phosphorylated Akt and Erk following VEGF treatment within 60 min. The histograms represent densitometric analysis of phosphorylated Akt and Erk. (B) Western blot analysis of phosphorylated Akt and Erk following VEGF treatment after 9 h and densitometric analysis of phosphorylated Akt and Erk. (C) Changes of miR‐20a and miR‐31 levels following VEGF treatment (9 h) in the presence or absence of LY294002 (50 μm). (D) Changes of mRNA level of TNFSF15 in response to VEGF treatment in the presence or absence of Akt inhibitor LY294002. (E) Changes of protein level of TNFSF15 in response to VEGF treatment in the presence or absence of Akt inhibitor LY294002. (F) Changes of miR‐20a and miR‐31 levels following VEGF treatment (9 h) in the presence or absence of Erk inhibitor U0126 (10 μm). (G) Changes of TNFSF15 mRNA level following VEGF treatment in the presence or absence of U0126. (H) Changes of protein level of TNFSF15 in response to VEGF treatment in the presence or absence of Erk inhibitor U0126. Data are mean ± SD. *P < 0.05; **P < 0.01; ***P < 0.001; Student's *t*‐test or one‐way ANOVA.

We investigated signaling pathways activated by VEGF and involving the upregulation of miR‐20a and miR‐31. First, we treated HUVECs cultures with VEGF (100 ng·mL^−1^) and determined the impact on a number of intracellular signaling molecules known to respond to VEGF. PI3K/Akt and Erk are pivotal signal transduction pathways related to EC proliferation and survival 25. We found by western blot analysis that Akt and Erk became activated within 5–10 min following VEGF treatment (Fig. 4A), and the effects remained detectable for as much as 9 h (Fig. 4B). We then treated the cells with PI3K‐Akt inhibitor LY294002 and found that the inhibitor blocked the expression of miR‐20a and miR‐31 (Fig. 4C). Inhibition of Akt phosphorylation by using LY294002 also resulted in VEGF‐induced downregulation of TNFSF15 mRNA level (Fig. 4D). Simultaneously, the expression of TNFSF15 in protein level was also downregulated (Fig. 4E). Additionally, we treated HUVECs cultures with Erk inhibitor U0126, and found that the inhibitor prevented VEGF‐stimulated expression of miR‐20a but not that of miR‐31 (Fig. 4F). U0126 treatment also prevented VEGF‐induced downregulation of TNFSF15 mRNA expression (Fig. 4G). In addition, the protein level of TNFSF15 was downregulated as well (Fig. 4H). These data indicate that VEGF activation of Akt and Erk led to upmodulation of miR‐20a and miR‐31, and coincidentally the downregulation of TNFSF15.

### miR‐20a and miR‐31 inhibit capillary‐tubule formation by HUVECs *in vitro*

**Figure 5:**
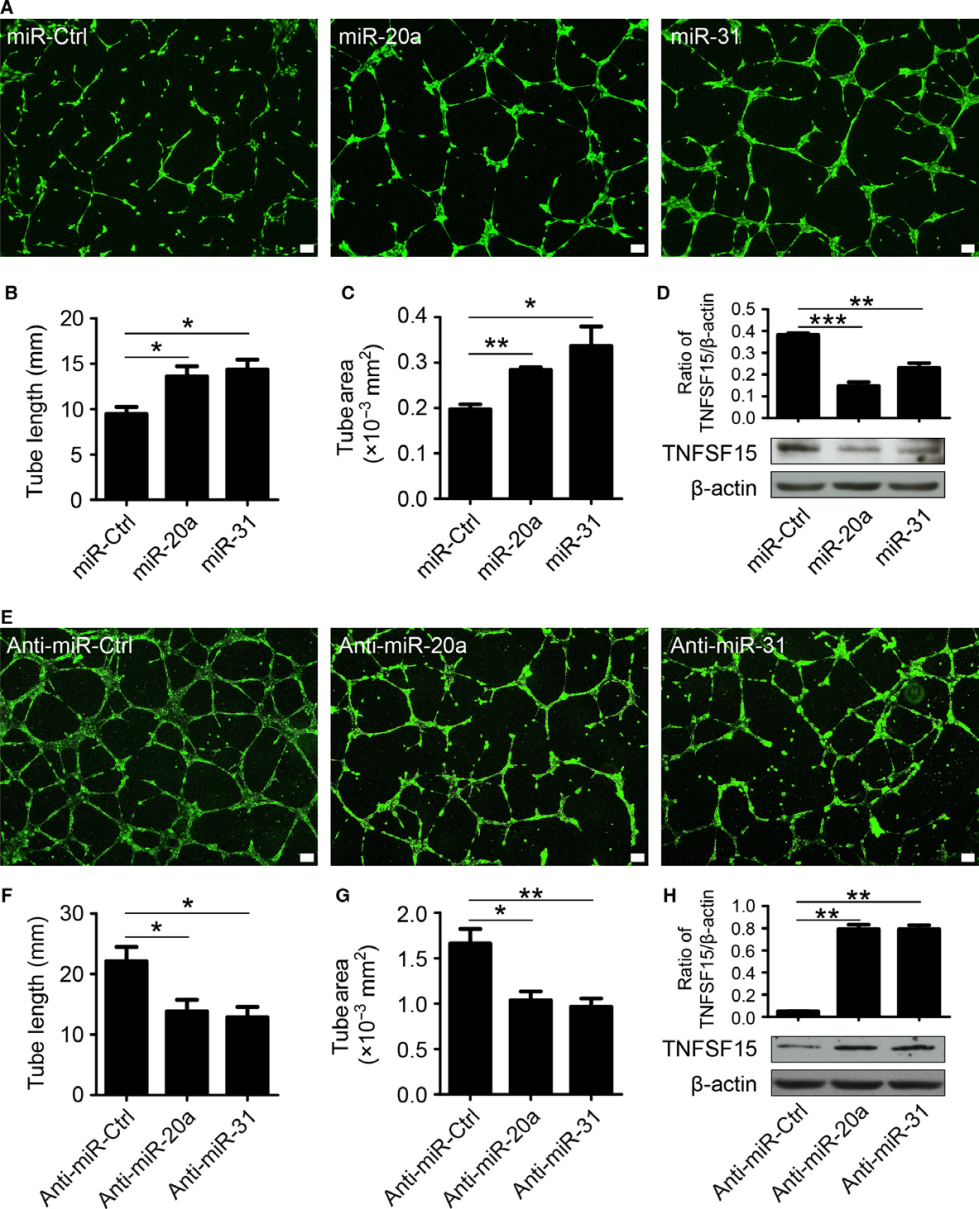
miR‐20a and miR‐31 inhibit capillary‐tubule formation by HUVECs *in vitro*. (A) Images of capillary‐like tubules formed by HUVECs transfected with miR‐Ctrl, miR‐20a, or miR‐31 mimics on Matrigel in 9 h after seeding; scale bar, 100 μm. (B) Average lengths and areas (C) of the capillary‐like tubules formed by miR‐Ctrl‐, miR‐20a‐, and miR‐31 mimics‐transfected cells. (D) Western blot analysis of TNFSF15 protein levels in transfected cells. Bar graphs represent quantitative analyses of western blot data. (E) Images of capillary‐like tubules formed by HUVECs transfected with anti‐miR‐Ctrl, anti‐miR‐20a, or anti‐miR‐31 on Matrigel in 9 h after seeding; scale bar, 100 μm. (F) Average lengths and (G) areas of the capillary‐like tubules formed by anti‐miR‐Ctrl‐, anti‐miR‐20a‐, and anti‐miR‐31‐transfected cells. (H) Western blot analysis of TNFSF15 protein levels in transfected cells. Bar graphs represent quantitative analysis of western blot data. Data are mean ± SD. *P < 0.05; **P < 0.01; ***P < 0.001; one‐way ANOVA.

We determined whether miR‐20a and miR‐31 were able to inhibit capillary‐tubule formation by HUVECs on a Matrigel coating. The cells were transfected with either miR‐20a, miR‐31, or miR‐control mimics, and seeded in culture‐wells coated with Matrigel. We found that miR‐20a‐ or miR‐31‐transfected cells were more capable of forming capillary‐like tubules compared to cells in the control group (Fig. 5A–C). Western blot analysis of cell‐associated TNFSF15 protein levels in these cultures indicated that TNFSF15 expression was inhibited (Fig. 5D). In addition, we transfected HUVECs with either anti‐miR‐20a, anti‐miR‐31, or anti‐miR‐control, and examined the abilities of these cells to form capillary‐like tubules. We found that anti‐miR‐20a or anti‐miR‐31 inhibited tubule formation in comparison with the effect of the anti‐miR‐control‐transfection (Fig. 5E–G). At the same time, cell‐associated protein levels of TNFSF15 were found to be much higher in anti‐miR‐20a‐ or anti‐miR‐31‐transfected cells compared with those in the control group (Fig. 5H). These data are consistent with the view that miR‐20a and miR‐31 can inhibit TNFSF15 gene expression, which in turn gives rise to stimulation of angiogenesis *in vitro*.

## Discussion

Vascular endothelial growth factor and TNFSF15 appear to be a pair of cytokines with counteractivities in the modulation of vascular homeostasis. VEGF has a central role in promoting angiogenesis under a variety of physiopathological conditions 26, whereas TNFSF15 is involved in the maintenance of the quiescence and stability of an established vasculature 1, 8, 27, 28. We reported previously that VEGF, whose gene expression is kept at low levels in normal tissues but highly upregulated in tissues where angiogenesis is taking place such as in cancer tissues, is able to inhibit the gene expression of TNFSF15, which in contrast to that of VEGF is prominent in normal tissues but diminishes in angiogenic tissues 8. In this study, we demonstrate that VEGF‐initiated downmodulation of TNFSF15 materializes through a mechanism that involves a number of miRNA molecules. Specifically, VFGF is able to stimulate the production of miR‐20a and miR‐31 in endothelial cells to eradicate TNFSF15 mRNA. Our study also reveals a number of signaling pathways involving Akt and Erk that are activated by VEGF in endothelial cells and directly lead to upregulation of the expression of miR‐20a and miR‐31.

Initially, through an inspection of the 3′‐UTR of the TNFSF15 gene transcript, coupled with data mining of the known microRNA pools, we identified 12 miRNA molecules in endothelial cells that might target TNFSF15. However, only two of them, namely miR‐20a and miR‐31, exhibited upregulated expression patterns in response to VEGF treatment of the cells. It was recently reported by other investigators that miR‐20a and miR‐31 are noticeably upregulated in human tumors such as gastric cancer, nonsmall cell lung cancer, prostate cancer, and colorectal cancer 29, 30, 31, 32, 33. Findings by these investigators are consistent with our earlier report that VEGF produced by ovarian cancer cells can inhibit the production of TNFSF15 by endothelial cells in tumor tissues 8, as well as our findings here that miR‐20a and miR‐31 are involved in VEGF‐stimulated downmodulation of TNFSF15 activities in angiogenic endothelial cells.

We find that Akt and Erk signaling pathways are responsible for mediating VEGF‐initiated downregulation of TNFSF15 gene expression are of interest for two reasons. First, the involvement of these signaling pathways may provide a window of opportunity to interfere in order to restore the endogenous antiangiogenesis activity of TNFSF15. Second, as these signaling pathways are generally involved in cell growth promotion, our findings suggest that other cytokines or growth factors that activate these pathways may also be able to downregulate TNFSF15 production, thus contribute to a destabilization of an established vasculature, facilitating blood vessel growth. Our data are supported by an earlier report that the Erk–Elk pathways are capable of regulating the miR‐17–92 cluster 34, of which miR‐20a belong.

Our findings suggest that miR‐20a and miR‐31 are potentially oncogenic as they cause a repression of the expression of TNFSF15 gene, which appears to be able to function as a gate‐keeper of neovascularization in cancers 8. This notion is supported by a previous study suggesting that miRNA‐31‐5p is implicated in tumor progression 35. Anti‐miRNA may be of use with regard to the development of antiangiogenic and anticancer approaches.

In summary, we identified two miRNA molecules, miR‐20a and miR‐31 that are able to target the TNFSF15 gene, preventing the latter to function as an inhibitor of angiogenesis. The production of these two miRNAs is stimulated by the Akt and Erk signals, which is activated by the angiogenic factor VEGF. This mode of action may be found in cancers, especially at the onset of early stages of tumorigenesis when the mature, stable vasculature of the involved tissue is to become destabilized and new blood vessel growth is to be initiated.

## Author contribution

HTD and HLL performed the experiments. HTD, HLL, and QZZ produced the figures. HTD, QZZ, and LYL designed the study, analyzed the data, and wrote the manuscript. BBZ, KZ, GCX, and XMP participated in discussion during the preparation of the manuscript. All authors had final approval of the submitted and published versions.
